# The Ethics of Dying: Deciphering Pandemic-Resultant Pressures That Influence Elderly Patients’ Medical Assistance in Dying (MAiD) Decisions

**DOI:** 10.3390/ijerph18168819

**Published:** 2021-08-21

**Authors:** Masud Khawaja, Abdullah Khawaja

**Affiliations:** 1 University of the Fraser Valley, Abbotsford, BC V2S 7M8, Canada; 2 University of British Columbia, Vancouver, BC V6T 1Z4, Canada; akhawa01@gmail.com

**Keywords:** COVID-19, bioethics, patient autonomy, beneficence, non-maleficence, access to resources, social support

## Abstract

The objective of medicine is to provide humans with the best possible health outcomes from the beginning to the end of life. If the continuation of life becomes unbearable, some may evaluate procedures to end their lives prematurely. One such procedure is Medical Assistance in Dying (MAiD), and it is hotly contended in many spheres of society. From legal to personal perspectives, there are strong arguments for its implementation and prohibition. This article intends to add to this rich discourse by exploring MAiD in the context of our current pandemic-ridden society as new pressures from social isolation and guilt threaten the autonomy of vulnerable elderly patients. Although autonomy is of chief importance, variables within our current context undermine otherwise independent decisions. Many older individuals are isolated from their social network, resulting in a decline in their mental health. Individuals in such a state are more likely to request a MAiD outcome. Furthermore, overwhelmed healthcare systems may not adequately address this state, which would normally have prompted a mental health intervention. The future of MAiD is far from settled and careful consideration must be given as new contexts come to light, such as those outlined in this paper.

## 1. Introduction

This paper illuminates new ethical concerns related to Medical Assistance in Dying (MAiD) through a bioethical lens amid current pandemic conditions. Specifically, in the current context, elderly populations have been affected by a myriad of new challenges that add complexity to MAiD discourse. Evolving MAiD legislation must comprehensively account for all the forces elderly patients experience when making end-of-life decisions. The failure to recognize new pressures threatens patient autonomy among this vulnerable population. To develop ethical sapience in this sensitive discussion, we examine several contributing factors, including current practices, availability of resources, social support, and patient autonomy. In the realm of traditional bioethics, a patient’s right to autonomous decision-making is central and must be respected and upheld. However, as Walker [1] explains, no decision is truly autonomous. The pandemic presented new waves of social isolation that impacted elderly populations tremendously. Further pressuring elderly patients was the much-discussed strain on medical resources throughout the past eighteen months. These pressures, though indirect, may obfuscate autonomous decision-making, which presents an ethical quandary for healthcare providers seeking to act in their patients’ best interests. In short, this paper reveals new points of discussion related to MAiD practices.

## 2. Current Viewpoints on MAiD

As the name suggests, MAiD calls upon health professionals to aid in a patient’s suicide either by prescribing life-ending drugs or through methods of active euthanasia [2]. For the purposes of this article, the distinction is unimportant, as the focus is on the factors motivating the decision to undertake the process, and not the process itself. The immediacy and finality of MAiD demands a narrow lens of analysis.

While Canada, the Netherlands, Switzerland, Belgium, and several US states have legally granted individuals the right to MAiD [3,4], its acceptance in the western world is not widespread. In several other countries, MAiD is a moral and legal “grey area” in which medical practice is at odds with the law, potentially signaling that practice shifts with public opinion, opinion moves with practice, or the two move together. For example, South Africa’s position on the matter is unclear; the practice is illegal but, in cases where MAiD was performed, there were no legal consequences [5]. Meanwhile, in New Zealand, MAiD is set to become legal in late 2021 with mixed opinions [6]. In Canada, the procedure is available to adults only, near death, suffering from a non-mental illness, with no recovery possible. However, in other countries, such as Belgium and the Netherlands, the procedure is available to minors and those suffering from an incurable mental illness [7]. Perception of whether one’s faculties have degraded to a point that warrants eligibility for MAiD is driven by worldview, perception, and stereotypes of ability [8]. This undoubtedly makes determining candidacy for the procedure difficult. The contention between societal or personal objections and legal performative duty adds to the complexity surrounding MAiD legislations.

The Belmont Report describes the need to protect those individuals whose ability to make autonomous decisions has diminished [9]. COVID-19 has added new stressors to all of society, but elderly individuals, who are already more likely to undertake MAiD, have faced unique challenges due to their frailty. Baroness Tanni Grey-Thompson [10] recognizes pressures stemming from family as well as mental illness as causes for concern when assessing MAiD requests. Constructing legislation without considering emerging contexts may result in poorer outcomes for these patients. The United Nations Educational, Scientific and Cultural Organization (UNESCO) Universal Declaration on Bioethics and Human Rights echoes and adds to this sentiment as it details the responsibility of legislators to uphold global medical ethics [11]. As we emerge from the depths of the COVID-19 pandemic, the tolls on mental health are undetermined, especially for candidates of MAiD. Research must be undertaken to fully understand the context surrounding further legislative advances.

The legalization of MAiD has been a long and arduous process due to moral arguments on both sides. In support of MAiD, arguments pertain to respect for patient autonomy and relief of suffering [12]. More recently, the argument for safe medical practice has also gained traction. MAiD envisages death in a safe and dignified manner that other methods of suicide cannot [12]. Alternatively, opposition to MAiD includes: the argument of lack of autonomy, the claim of existing alternatives, inequalities, and the antagonism between medically assisted death and palliative care [13]. Given these contrasting arguments, adoption from all stakeholders lags even when it is legalized. This includes healthcare providers, who may be legally required to provide MAiD, despite personal moral objections.

**Figure 1:**
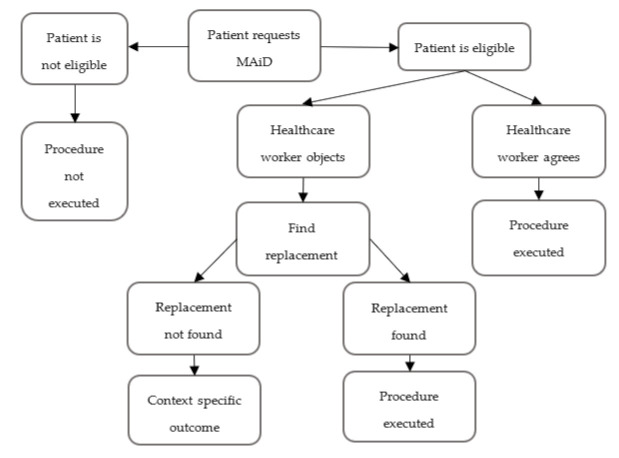
MAiD Decision Tree.

Healthcare practitioners who are mandated to carry out patients’ wishes and respect their autonomy may find themselves betraying their own beliefs. In their study of nursing students, Ozcelik et al. [14] found that one’s moral standing and religious affiliation can affect their perception of MAiD. In Canada, religious freedoms are primogeniture in the eyes of the law; it would be a charter violation to require a healthcare worker to assist in MAiD if it conflicts with their religious beliefs. However, as the pandemic worsened, some governments chose to limit religious freedoms by restricting all religious groups’ ability to hold gatherings, etc. Many Canadians may believe that restricting charter rights is appropriate given the pandemic situation. Whether this practice extends to a medical context is unclear. Perhaps healthcare providers could be permitted to opt out of performing the procedure and replaced with someone who is not morally opposed. However, medical resources and staff remain scarce in many countries, so there is no guarantee of this possibility. The correct solution may remain unclear until long after this pandemic is over. A possible decision tree for this situation is illustrated in Figure 1.

Healthcare systems have been ostensibly altered in the wake of COVID-19. As prerequisites for MAiD, Canadian standards require intense suffering and no hope of recovery. The pandemic conditions have added to an already contentious issue, as COVID-19 complications in immunocompromised patients could result in candidacy for MAiD. Many patients view MAiD as an increasingly viable alternative to passive euthanasia. Healthcare systems that have been further burdened by pandemic-related illnesses have denied patients the best care otherwise possible. MAiD thus offers individuals the option to die with dignity on their terms rather than slowly succumbing to illness, disease, or old age.

## 3. Respecting Patients’ Decisions

Crucial to decision-making, the principle of autonomy is among the most fundamental tools of medical ethics. In opposition to paternalism, patient autonomy seeks to ensure the right of a patient to make their own decisions while providing realistic information without undue influence [15]. Thus, patient autonomy must be prioritized in any MAiD-related decision, regardless of one’s position on this matter. However, patients make decisions based on several factors, including their education, family, religious values, culture, and past experiences, all of which influence autonomy. Society also shapes perceptions surrounding disability regarding how one may be treated or discriminated against [16]. Where manageable, a patient may see divergent needs as debilitating, and societal stigma may reinforce this idea, resulting in an avoidable MAiD decision. As medicine moves further away from paternalism, patient autonomy is increasingly being prioritized above all else. Supporters of MAiD contend that the principle of autonomy allows an individual to make all medical choices in their life, including the choice to end it. This aligns with the modernized patient-centered care [17], which has evolved to reflect autonomy in place of traditional paternalistic practice.

In the context of MAiD, however, patient autonomy remains ill-defined. Should a patient decide that MAiD is a preferable outcome, physicians may not have the authorization to act accordingly. As previously stated, much of the world does not legally allow this procedure. Additionally, Appelbaum [18] found physicians expressed concerns regarding the role of mental health in MAiD decision making. Mental health concerns include whether the patient is deciding without undue influence, and if the patient is of sound mind. As COVID-19 has been attributed to a large increase in depression and anxiety [19], current conditions may influence patients’ decisions in a way that is not aligned with the spirit of autonomy. Declining mental health may also result in patients refusing beneficial treatment and exercising a right-to-die option [20]. Determining the true desires of a patient can be difficult, even in optimal conditions. Today, this process may prove more precarious for overworked, burnt-out healthcare staff in systems greatly affected by COVID-19. Failure to adequately consider a patient’s mental state may result in MAiD decisions that could have been avoided in favor of mental health interventions. Physicians may also hesitate to perform the procedure based on personal, moral, or spiritual objections, as well as some concern that the procedure is “out of scope” for medical professionals [21].

Patients must be given complete information, including a thorough discussion regarding diagnosis, treatment options, and prognosis, if they are to make informed decisions and exercise autonomy. These discussions must take place in a manner that can be understood by the patient while respecting their unique cultural and educational background. The ability to provide comprehensive information that respects all aspects of autonomy and conforms to cultural values may be affected by limited resources and the availability of healthcare staff. When a patient is likely to pass away from COVID-19 or otherwise, and their treatment will consume limited available resources, pressures could sway an overworked healthcare professional to provide incomplete information and nudge the patient towards MAiD in a way that violates autonomy. For these reasons, a balance must be struck between medical evidence, patients’ beliefs, and patients’ attitudes towards life and death.

## 4. Lack of Social Support

Social support is another important factor that practitioners must concern themselves with, especially in relation to the elderly population since the onset of the pandemic. Members of this group may have lost their spouse, likely do not have parents, and other familial support that is available to a younger demographic. Elderly patients in hospitals and long-term care facilities are typically among the most ill in society, and thus most likely to consider MAiD. In the United States, two out of three patients who opt for MAiD are over 65, and the median age of medically assisted death is 74 [22]. Physical distancing protocols, stay-at-home orders, travel restrictions, and widespread cancellations of in-person school, work, and events have all contributed to increased isolation in most of our lives. Armitage and Nellums [23] found that pandemic-induced isolation has been particularly severe for the elderly. Most of these people live alone rather than with family or roommates, making social isolation more extreme [24]. Furthermore, many elderly people rely on activities outside their homes for connectivity, such as daycare venues, community centers, and places of worship [25], which have been halted. Older people also tend to lack literacy concerning technology. While many people use technology to maintain relationships, the elderly are less likely to utilize this tool due to technological incompetence [26]. Finally, many elderly people and their loved ones chose to voluntarily increase isolation measures as a means of self-preservation, given their heightened risk of severe complications from COVID-19. For example, a nurse who once provided support for an elderly parent might recognize that the risk of viral transmission is too high and decide to stop visiting, even if not required to do so according to public health orders. 

Like most of us, elderly people have experienced an overall decreased quality of life since the onset of the pandemic [27]. However, the intense loneliness of social isolation has been more severe for those in hospitals and long-term care (LTC) facilities. Most such services greatly restricted visitors or completely quarantined to protect patients and staff. Aside from limited virtual interactions, many were cut off from their loved ones. One physician in an LTC facility described the profound isolation of this population: “my patients have become prisoners in their one-bedroom homes, isolated from each other and the outside world” [26]. This loneliness raises additional concerns as it is a known risk factor for poor health outcomes, including anxiety, depression, malnourishment, and worsening dementia [26]. Briguglio et al. [27] also observed that isolation caused diminished resilience in elderly people, which may have worsened age-associated conditions.

Social support is generally defined as the availability of people whom one can rely upon and experience care and love [28]. According to Shroepfer [29], social support is a positive aspect of relationships wherein individuals reciprocate instrumental, informational, or emotional support they need to remain healthy. A lack of social support is viewed as a barrier to the best health outcomes [30]. The need for social support is heightened when it comes to elderly populations as it mediates loneliness and depression [28]. With social support largely stripped away due to COVID-19 safety measures, we can assume that rates of loneliness and depression have increased dramatically among the elderly. Many such restrictions were enacted to protect this physically vulnerable demographic, as was certainly necessary. However, the diminished social support has adversely and disproportionately impacted the elderly. Further, research shows that people with low social support levels have a higher risk of mortality when compared to people who have more supportive networks [31].

Studies confirm that positive social support results in health-enhancing and preventative health behaviors among elders [32]. Terminally ill individuals considering MAiD have fewer, or lower quality social supports [33]. This knowledge must be synthesized into decision-making when we consider the implications of MAiD, given the isolation due to the pandemic. Elderly patients may request MAiD because of the loneliness associated with current circumstances, whether this reasoning is conscious or unconscious. This possibility is twofold. Firstly, we know that the physical health of elderly people has declined because of social isolation, including worsening dementia and diminished resilience to a myriad of age-related health conditions [25,27]. Secondly, we can assume that mental health has plummeted for those elderly people who have experienced isolation and profound loneliness due to the pandemic. To prevent such influence, social support should be involved in end-of-life decisions whenever possible. This is especially important considering that a lack of social support is one predictor that a patient will request MAiD [34], indicating that those without it will be more likely to consider ending their life prematurely.

A lesser acknowledged and examined relationship between social support and MAiD is the indirect social control that dictates a sense of responsibility toward loved ones that those dying feel. It leads patients to consider a hastened death as a health behavior that would negatively impact their loved ones [29], both emotionally and financially. For example, one patient expressed concerns about her much younger husband’s financial welfare: “that’s what bothered me most about the whole thing… more than me dying, was leaving my family behind with all the bills and what were they going to do, and how is he [husband] going to get along without my pension” [30]. This reveals that humans in general feel responsible for the loved ones they leave behind. However, mental health impairment constitutes a state of mind in which they may not be qualified to judge their final decision of MAiD, whereas treatment to improve outlook can be provided.

Thus, to proceed with MAiD despite a lack of social support presents ethical and moral dilemmas. Social support, such as family, may provide more holistic insight on the patient’s journey to the MAiD decision. For the best outcomes when treating elderly patients, it is said that decisions should be made over a series of conversations that include the healthcare provider, the patient, and the family [35]. In this way, the patient is supported when making difficult decisions. A lack of social support may, therefore, be considered an undue influence stemming from the pandemic circumstances, thus clouding the decision of the patient. A patient’s judgment must never be clouded by depression or isolation from loved ones. If a patient’s mental health diminishes due to external factors, such as pandemic-related isolation, their autonomy when making this decision is clearly threatened. However, when MAiD is the true desire of a terminal patient, they have the support of their loved ones, and it is legally permissible, it may be made available to the patient.

## 5. Limited Access to Resources

Accessing adequate medical resources has been an ongoing challenge throughout the COVID-19 pandemic. For example, the supply of personal protective equipment (PPE) for healthcare workers has been inadequate at times [36]. Similarly, at the height of the pandemic, ventilators also became a scarce resource [36], affecting access to treatment for COVID-19 patients, in addition to patients with other ailments requiring the device. As such, chronically ill patients, and elderly patients in particular, may experience pressure to relieve resources. Older patients, who have retired and are less active in their community, may feel this type of pressure that a younger patient would be immune to. It would, in such circumstances, be a severe degradation of patient autonomy to be pressured into making a MAiD decision. One facet of justice in bioethics pertains to the need for equitable distribution of available resources [37]. Thus, in line with bioethics, resources must be optimally distributed to elderly people and younger, healthier patients. Careful consideration must take place when making decisions related to equitable access to resources. In this way, no one will be disadvantaged unfairly, and traditional bioethics will be upheld.

Of all the resources required to keep healthcare systems operational, healthcare workers are the most important. However, the pandemic has been extremely taxing on this human resource. According to the World Health Organization (WHO), more than 100,000 healthcare workers have died from complications of the disease [38], while innumerable others have suffered immense consequences. Deaths aside, the most significant toll has been on the mental and emotional well-being of healthcare workers. The pressure on them has been monumental. Every day, they must decide “how to allocate scant resources to equally needy patients, how to balance their own physical and mental healthcare needs with those of patients, how to align their desire and duty to patients with those to family and friends, and how to provide care for all severely unwell patients with constrained or inadequate resources” [39]. This pressure was compounded by unprecedented staff shortages, as healthcare workers were especially at high risk of contracting the virus themselves. As a result, extreme exhaustion, burnout, workplace injuries, post-traumatic stress disorder, anxiety, and depression became widespread [40]. While vaccination efforts have begun to alleviate some of this stress, the emotional toll on healthcare workers will likely persist for years to come. As a result, some healthcare providers may find it difficult to display the same high-level standard of care and empathy, further jeopardizing the dignity usually afforded to patients near death. Regrettably, issues relating to resource availability may disproportionately affect the elderly, as inequities in medicine for the elderly have been well documented over time [41].

The unavailability of hospital resources affects end-of-life care options. MAiD may be presented in cases that lack palliative care options to spare a patient from a painful and undignified death. However, this is generally supposed to be reserved for those patients in constant pain, with no hope of recovery, and facing death. Early in the pandemic, Yang et al. [42] found that older individuals were at an increased risk of death due to COVID-19. The justification of MAiD cannot be based solely on limited resources, as this would contradict accepted medical ethics, such as beneficence and non-maleficence. In addition, there is apprehension that palliative care quality and standards may suffer over time in countries and regions where MAiD is legal. The general effort toward treating elderly sufferers may decline over time if the option to end the lives of COVID-19 patients or sufferers of other ailments is available. Extending traditional thoughts of justice includes acknowledging the potential for some to be exploited, as well as inequitable resource allocation impacting the historically disadvantaged people [43]. Palliative care options may only include MAiD if a patient is near death with little hope of survival, on the condition that they are fully informed of all options available to them to ensure personal autonomy.

## 6. Ensuring Best Patient Outcomes

The ethical principle of beneficence stipulates that a physician must act in the best interest of their patients. However, because patients are often poorly informed about the nuances of medical treatment, beneficence is usually exercised by the physician based on what they think will work for the patient. Decisions regarding what is best for others will undoubtedly leak into the thought process or discussion. When resources are unavailable, that will weigh heavily on the physicians, as well as the patients. Jordan et al. [44] found that older COVID-19 patients admitted to the ICU typically stay about two weeks. This may be insufficient time to adequately weigh all the factors and decide what is best for the patient.

The ethical principle of non-maleficence dictates that a physician must do no harm when caring for patients [45]. However, what constitutes harm to the patient may be unclear in cases concerning MAiD. Various decisions made by physicians may cause some element of harm to a patient. Thus it is essential to view such outcomes in relation to more serious harms. The principle of non-maleficence seeks to mitigate the harm caused by assuming that the benefit of the intervention outweighs the potential harm, which is subject to personal opinions. In a strictly literal consideration of non-maleficence of doing no harm, MAiD appears to cause the ultimate harm to an individual as it ends life prematurely.

Most, but not all, pain is now manageable in cases of disease, due to the progression of modern medicine [46]. However, some argue that current pain management techniques in terminal illnesses are still insufficient to justify a ban on MAiD [13]. Regardless, COVID-19 has opened our eyes to the possibility of a crippled medical system that leaves patients to die without the best treatment possible, but rather with only the available treatment. In such situations, it is possible to do what may be best for the patient while harming them least: allowing them to undergo MAiD.

## 7. Conclusions

Where MAiD has been legislated, there is an expectation that the patient will act autonomously. However, as outlined, mental health declines impact decision-making in this regard. Physical distancing and social isolation protocols largely eliminated social support for most hospital and long-term care patients [47], as well as for the larger elderly population [48]. In such situations, more patients, especially older ones, may choose to exercise a MAiD option prematurely. It is contended that MAiD should continue to be reserved only for patients that are terminally ill, with zero hope of recovery [49]. While the circumstances surrounding MAiD may allow for abuse, this procedure does have its place in some contexts. Family members of patients who had undergone MAiD generally considered the experience to be positive [50]. As alluded to earlier, when the procedure is used to release an individual from suffering, with no hope of recovery, there may be value. Given the unique challenges faced by elderly patients, careful assessment of mental health and motivation should be performed, especially as it relates to social isolation.

In a situation where healthcare workers observe that a lack of social support is influencing the patient’s decision, they may object to performing the procedure. However, when the patient has the final say, healthcare workers are left with no choice. While patients may decide if MAiD aligns with their moral and spiritual beliefs, this choice is not given to healthcare workers who are expected to execute the order upon request. After Canada passed the assisted dying legislation in 2016, this was the consensus among interviewed nurses [51]. However, when adjusting medical ethics to suit pandemic conditions, autonomy must be paired with beneficence, to honor the desires of the patient whilst ensuring the best outcomes.

The strain on resources raises additional ethical quandaries for the treatment of the elderly. In pandemic conditions, there are concerns that those patients may not receive the dignity and comfort that is expected under usual circumstances. This is evident when examining the havoc that COVID-19 has wreaked on medical systems around the world. Medical resources have been pushed to their absolute limits [52], while healthcare workers have faced immense pressure leading to burnout and depression [53]. Considering these revelations, elderly patients may feel as though they are taking up resources unnecessarily. They may also fear a painful death if high-quality care is unavailable. Patients must not resort to MAiD out of guilt or fear. To perform MAiD on a patient for these reasons would be a breach of medical non-maleficence. Current legislation fails to account for these circumstances, leaving a void.

Prolonged suffering, extinguished mental capacity, and complete loss of dignity are all grounds to allow for a medically aided death [54]. However, the innate desire of medicine is to preserve life and restore health. As such, many believe that medicine should strive to preserve life at all costs. Given the risks inherent in MAiD, should it continue to be legislated? Though some countries have legalized MAiD, many ethical questions surrounding the practice remain unresolved. MAiD is fraught with dangers and risks in its turbulent future, and the aftermath of the COVID-19 pandemic only complicates the matter further. The threat COVID-19 has posed to the ballooning elderly population can never be forgotten. This demographic is a much more likely candidate for MAiD moving forward. Pressures on the healthcare system from this large segment of the population could provide an impetus for the widespread passage of MAiD legislation throughout the world. It is acknowledged that the discussion within this article is bound to a Western context, which limits its generalizability to other regions. Future studies may seek answers to the ever-changing perception of MAiD from patients and healthcare workers around the world as we near the end of the pandemic. As we all become more intimately aware of our medical systems and the pressures they face, public perception of MAiD and our empathy towards healthcare workers will no doubt change.

## References

[1] Walker R.L. (2008). Medical ethics needs a new view of autonomy. J. Med. Philos..

[2] Li M., Watt S., Escaf M., Gardam M., Heesters A., O’Leary G., Rodin G. (2017). Medical assistance in dying—Implementing a hospital-based program in Canada. N. Engl. J. Med..

[3] Emanuel E.J., Onwuteaka-Philipsen B.D., Urwin J.W., Cohen J. (2016). Attitudes and practices of euthanasia and physician-assisted suicide in the United States, Canada, and Europe. JAMA.

[4] Pope T.M. (2018). Legal history of medical aid in dying: Physician assisted death in US courts and legislatures. NML Rev..

[5] Jacobs R.K., Hendricks M. (2018). Medical students’ perspectives on euthanasia and physician-assisted suicide and their views on legalising these practices in South Africa. S. Afr. Med. J..

[6] Nie L., Smith-Han K., Iosua E., Walker S. (2021). New Zealand medical students’ views of euthanasia/assisted dying across different year levels. BMC Med. Educ..

[7] Buote L.C., Wada K., Russell-Mayhew S., Feldstain A. (2021). Maid in Canada: Controversies, guidelines, and the role of psychologists in relation to Bill C-14. Can. Psychol..

[8] Johnson H.M. (2003). Unspeakable conversations. Moral Issues Glob. Perspect..

[9] U.S Department of Health & Human Services. The Belmont Report. https://www.hhs.gov/ohrp/regulations-and-policy/belmont-report/read-the-belmont-report/index.html.

[10] Grey-Thompson T. Speech by Baroness Tanni Grey-Thompson to Isle of Man House of Keys. Life Charity. https://lifecharity.org.uk/news-and-views/speech-by-baroness-tanni-grey-thompson-to-isle-of-man-house-of-keys/.

[11] United Nations Educational, Scientific and Cultural Organization Universal Declaration on Bioethics and Human Rights. http://portal.unesco.org/en/ev.phpURL_ID=31058&URL_DO=DO_TOPIC&URL_SECTION=201.html.

[12] Dugdale L.S., Lerner B.H., Callahan D. (2019). Pros and Cons of Physician Aid in Dying. Yale J. Biol. Med..

[13] Barutta J., Vollmann J. (2015). Physician-assisted death with limited access to palliative care. J. Med. Ethics.

[14] Ozcelik H., Tekir O., Samancioglu S., Fadiloglu C., Ozkara E. (2014). Nursing students’ approaches toward euthanasia. OMEGA J. Death Dying.

[15] Taylor J.S. (2018). Introduction: Autonomy in healthcare. HEC Forum.

[16] Shakespeare T. (2019). When the political becomes personal: Reflecting on disability bioethics. Bioethics.

[17] Bokhour B.G., Fix G.M., Mueller N.M., Barker A.M., LaVela S.L., Hill J.N., Solomon J.L., Lukas C.V. (2018). How can healthcare organizations implement patient-centered care? Examining a large-scale cultural transformation. BMC Health Serv. Res..

[18] Appelbaum P.S. (2016). Physician-assisted death for patients with mental disorders—Reasons for concern. JAMA Psychiatry.

[19] Ettman C.K., Abdalla S.M., Cohen G.H., Sampson L., Vivier P.M., Galea S. (2020). Prevalence of depression symptoms in US adults before and during the COVID-19 pandemic. JAMA Netw..

[20] Sulmasy D.P., Finlay I., Fitzgerald F., Foley K., Payne R., Siegler M. (2018). Physician-assisted suicide: Why neutrality by organized medicine is neither neutral nor appropriate. J. Gen. Intern. Med..

[21] Vekaria B., Overton C., Wisniowski A., Ahmad S., Aparicio-Castro A., Curran-Sebastian J., Eddleston J., Hanley N., House T., Kim J. (2020). Hospital length of stay for COVID-19 patients: Data-driven methods for forward planning. Res. Sq..

[22] Death with Dignity Frequently Asked Questions. https://deathwithdignity.org/learn/faqs/.

[23] Armitage R., Nellums L.B. (2020). COVID-19 and the consequences of isolating the elderly. Lancet Public Health.

[24] Kotwal A.A., Holt-Lunstad J., Newmark R.L., Cenzer I., Smith A.K., Covinsky, K (2021). E.; Perissinotto, C.M. Social isolation and loneliness among San Francisco Bay Area older adults during the COVID-19 shelter-in-place orders. J. Am. Geriatr. Soc..

[25] Eghtesadi M. (2020). Breaking social isolation amidst COVID-19: A viewpoint on improving access to technology in long-term care facilities. J. Am. Geriatr. Soc..

[26] Kasar K.S., Karaman E. (2021). Life in lockdown: Social isolation, loneliness and quality of life in the elderly during the COVİD-19 pandemic: A scoping review. Geriatr. Nurs..

[27] Briguglio M., Giorgino R., Dell’Osso B., Cesari M., Porta M., Lattanzio F., Peretti G.M. (2020). Consequences for the elderly after COVID-19 isolation: FEaR (frail elderly amid restrictions). Front. Psychol.

[28] Wang X. (2016). Subjective well-being associated with size of social network and social support of elderly. J. Health Psychol..

[29] Shroepfer T.A. (2018). Social relationships and their role in the consideration to hasten death. Gerontologist.

[30] Holt-Lunstad J., Uchino B.N., Glanz K., Rimer B.K., Viswanath K. (2015). Social support and health. Health Behavior: Theory, Research, and Practice.

[31] Wright K. (2016). Social networks, interpersonal social support, and health outcomes: A health communication perspective. Front. Commun..

[32] Potts M.K., Hurwicz M.L., Goldstein M.S., Berkanovic E. (1992). Social support, health-promotive beliefs, and preventive health behaviors among the elderly. J. Appl. Gerontol..

[33] Breitbart W., Rosenfeld B.D., Passik S.D. (1996). Interest in physician-assisted suicide among ambulatory HIV-infected patients. Am. J. Psychiatry.

[34] Smith K.A., Harvath T.A., Goy E.R., Ganzini L. (2015). Predictors of pursuit of physician-assisted death. J. Pain Symptom Manag..

[35] Kirchhoff K.T., Faas A.I. (2007). Family support at end of life. AACN Adv. Crit. Care.

[36] Ranney M.L., Griffeth V., Jha A.K. (2020). Critical supply shortages—The need for ventilators and personal protective equipment during the Covid-19 pandemic. NEJM.

[37] Johnstone M.J. (2019). Bioethics: A Nursing Perspective.

[38] World Health Organization Director-General’s Opening Remarks at the World Health Assembly-24 May 2021. https://www.who.int/director-general/speeches/detail/director-general-s-opening-remarks-at-the-world-health-assembly---24-may-2021.

[39] Greenberg N., Docherty M., Gnanapragasam S., Wessely S. (2020). Managing mental health challenges faced by healthcare workers during Covid-19 pandemic. BMJ.

[40] Greenberg N. (2020). Mental health of health-care workers in the COVID-19 era. Nat. Rev. Nephrol..

[41] Kilaru A.S., Gee R.E. (2020). Structural ageism and the health of older adults. JAMA.

[42] Yang X., Yu Y., Xu J., Shu H., Xia J., Liu H., Wu Y., Zhang L., Yu Z., Fang M. (2020). Clinical course and outcomes of critically ill patients with SARS-CoV-2 pneumonia in Wuhan, China: A single-centered, retrospective, observational study. Lancet Respir. Med..

[43] Gillon R. (2020). Raising the profile of fairness and justice in medical practice and policy. J. Med. Ethics.

[44] Jordan R.E., Adab P., Cheng K.K. (2020). Covid-19: Risk factors for severe disease and death. BMJ.

[45] Andersson G.B., Chapman J.R., Dekutoski M.B., Dettori J., Fehlings M.G., Fourney D.R., Weinstein J.N. (2010). Do no harm: The balance of “beneficence” and “non-maleficence”. Spine.

[46] Ali A., Arif A.W., Bhan C., Kumar D., Malik M.B., Sayyed Z., Ahmad M.Q. (2018). Managing chronic pain in the elderly: An overview of the recent therapeutic advancements. Cureus.

[47] Hwang T.J., Rabheru K., Peisah C., Reichman W., Ikeda M. (2020). Loneliness and social isolation during the COVID-19 pandemic. Int. Psychogeriatr..

[48] Plagg B., Engl A., Piccoliori G., Eisendle K. (2020). Prolonged social isolation of the elderly during COVID-19: Between benefit and damage. Arch. Gerentol. Geriatr..

[49] Government of Canada (2020). Medical Assistance in Dying. https://www.canada.ca/en/health-canada/services/medical-assistance-dying.html.

[50] Holmes S., Wiebe E., Shaw J., Nuhn A., Just A., Kelly M. (2018). Exploring the experience of supporting a loved one through a medically assisted death in Canada. Can. Fam. Physician.

[51] Pesut B., Thorne S., Schiller C.J., Greig M., Roussel J. (2020). The rocks and hard places of MAiD: A qualitative study of nursing practice in the context of legislated assisted death. BMC Nurs..

[52] Emanuel E.J., Persad G., Upshur R., Thome B., Parker M., Glickman A., Zhang C., Boyle C., Smith M., Phillips J.P. (2020). Fair allocation of scarce medical resources in the time of Covid-19. N. Engl. J. Med..

[53] Denning M., Goh E.T., Tan B., Kanneganti A., Almonte M., Scott A., Kinross J. (2021). Determinants of burnout and other aspects of psychological well-being in healthcare workers during the COVID-19 pandemic: A multinational cross-sectional study. PLoS ONE.

[54] Shukla R. (2016). Passive euthanasia in India: A critique. Indian J. Med. Ethics.

